# (Na, Zr) and (Ca, Zr) Phosphate-Molybdates and Phosphate-Tungstates: II–Radiation Test and Hydrolytic Stability

**DOI:** 10.3390/ma16030965

**Published:** 2023-01-20

**Authors:** M. E. Karaeva, D. O. Savinykh, A. I. Orlova, A. V. Nokhrin, M. S. Boldin, A. A. Murashov, V. N. Chuvil’deev, V. A. Skuratov, A. T. Issatov, P. A. Yunin, A. A. Nazarov, M. N. Drozdov, E. A. Potanina, N. Y. Tabachkova

**Affiliations:** 1 Physical and Technical Research Institute, Lobachevsky State University of Nizhny Novgorod, Nizhny Novgorod 603022, Russia; 2 G.N. Flerov Laboratory of Nuclear Reactions, Joint Institute of Nuclear Research, Dubna 141980, Russia; 3 Institute of Nuclear Physics and Engineering, National Research Nuclear University MEPhI (Moscow Engineering Physics Institute), Moscow 115409, Russia; 4 Department of Nuclear Physics, Dubna State University, Dubna 181982, Russia; 5 International Department of Nuclear Physics, New Materials and Technologies, The Faculty of Physics and Technology, Gumilov Eurasian National University, Nur-Sultan 010000, Kazakhstan; 6 Laboratory of Nuclear Processes, Nuclear Physics Department, The Institute of Nuclear Physics, Almaty 050032, Kazakhstan; 7 Laboratory of Diagnostics of Radiation Defects in Solid State Nanostructure, Institute for Physics of Microstructure, Russian Academy of Science, Nizhniy Novgorod 603950, Russia; 8 Center Collective Use “Materials Science and Metallurgy”, National University of Science and Technology “MISIS”, Moscow 119991, Russia; 9 Laboratory “FIANIT”, Laser Materials and Technology Research Center, A.M. Prokhorov General Physics Institute of the Russian Academy of Sciences, Moscow 119991, Russia

**Keywords:** NASICON, NZP, ceramics, spark plasma sintering, hydrolytic stability, radiation resistance

## Abstract

This paper introduces the results of hydrolytic stability tests and radiation resistance tests of phosphate molybdates and phosphate tungstates Na_1−x_Zr_2_(PO_4_)_3−x_(XO_4_)_x_, X = Mo, W (0 ≤ x ≤ 0.5). The ceramics characterized by relatively high density (more than 97.5%) were produced by spark plasma sintering (SPS) of submicron powders obtained by sol–gel synthesis. The study focused on hydrolytic resistance of the ceramics in static mode at room temperature. After 28 days of testing in distilled water, the normalized leaching rate was determined. It was found that the ceramics demonstrated high hydrolytic resistance in static mode: the normalized leaching rates for Mo- and W-containing ceramics were 31·10^−6^ and 3.36·10^−6^ g·cm^−2^·day^−1^, respectively. The ceramics demonstrated high resistance to irradiation with 167 MeV Xe^+26^ multiple-charged ions at fluences ranging from 1·10^12^ to 6·10^13^ cm^−2^. The Mo-containing Na_0.5_Zr_2_(PO_4_)_2.5_(XO_4_)_0.5_ ceramics were shown to have higher radiation resistance than phosphate tungstates. Radiation was shown to trigger an increase in leaching rates for W and Mo in the crystal structure of NZP ceramics.

## 1. Introduction

The NaZr_2_(PO_4_)_3_ compounds (NZP type) are among the most promising materials that can be used as matrices to immobilize highly active components of high-level radioactive waste (HLW). As noted in Part I hereof, such compounds meet requirements as to radiation resistance and hydrolytic stability [1,2,3,4,5,6,7,8,9,10,11,12,13,14,15]. Ceramics with an NZP structure can be quite effective at binding W and Mo into stable crystalline compounds where W and Mo can partially replace P. NZP ceramics may be used to immobilize Mo- and W-containing fractions of HLW [1,16,17,18,19,20,21,22,23,24].

One of the most promising methods for obtaining specimens of mineral-like ceramics is Spark Plasma Sintering (SPS), a new method of rapid hot pressing [25,26,27,28,29,30,31,32,33,34,35,36,37]. Ceramics are sintered in graphite dyes and heated by passing a high-powered millisecond pulsed current through them [25]. During sintering, specimens are subjected to uniaxial pressure, which allows for the high relative density of ceramics [4,25,26,27,28,29,30,31,32,33,34,35,36,37], without any fusible additives that are often added to powders to accelerate sintering (see [38]). A literature review shows that ceramics obtained by SPS are characterized by higher relative density and a fine-grained microstructure compared to ceramics obtained by conventional sintering of pre-pressed powders [4,28]. High heating rates, low sintering temperatures, and a short process time help minimize, if necessary, the dissociation of hazardous elements from the ceramic surface. Ceramics obtained by SPS have high radiation resistance and hydrolytic stability [4,39,40,41,42,43,44,45,46,47,48,49,50,51,52]. The efficiency of using SPS to obtain promising materials for nuclear power engineering was described in many key papers (see for example [46,53,54,55,56,57,58,59,60,61,62,63,64,65,66,67,68,69,70,71,72,73,74]). Currently, there are research papers on the process of obtaining NZP ceramics by SPS [4,49,75,76,77]. This allows for SPS to be considered a promising method of obtaining ceramic matrices to immobilize HLW [4,39,40,41,42,43,44,45,46,47,48,49,50,51,52,76,77].

Part I herein describes the crystal structure, microstructure, phase composition, and properties of phosphate molybdates and phosphate tungstates Na_1−x_Zr_2_(PO_4_)_3−x_(XO_4_)_x_ (NZP type) and Ca_1−x_Zr_2_(PO_4_)_3−x_(XO_4_)_x_ (CZP type). Part II herein studies the hydrolytic and radiation resistances of NZP ceramics containing various concentrations of Mo and W in their crystal structures. Particular attention is paid to compounds with a high content of Mo and W (x = 0.4, 0.5).

## 2. Materials and Methods

The Na_1−x_Zr_2_(PO_4_)_3−x_(XO_4_)_x_ solid solutions, having X = Mo, W, and x = 0.1, 0.2, 0.3, 0.4, 0.5 were the targets of this research. The compounds were synthesized using the sol–gel method. The ceramics were sintered from powders obtained by SPS using Dr. Sinter™ SPS-625 (SPS SYNTEX^®^, Kanagawa, Japan). A detailed description of the synthesis and sintering modes can be found in Part I.

The surface of the specimens, after sintering, contained residual carbon (graphite), which formed as a result of interaction between the ceramic specimens and a graphite dye wall and graphite foil. The ceramic specimens had very low crack resistance (see Part 1), which often led to micro-cracks during mechanical grinding of the specimens. To avoid cracks and remove carbon, the specimens were annealed in air at 700 °C for 2 h. After annealing, no residual carbon was detected on the surface of the specimens.

The XRD analysis of the irradiated ceramics was performed using a Bruker^®^ D8 Discover™ X-ray diffractometer in the symmetric Bragg–Brentano geometry. The microstructure of powders and ceramics was analyzed using a Jeol^®^ JSM-6490 scanning electron microscope (SEM) (Jeol Ltd., Tokyo, Japan) with an Oxford Instruments^®^ INCA 350 EDS microanalyzer (Oxford Instruments pls., Abingdon, UK). The methods used are described in Part I hereof.

The hydrolytic stability of the ceramic specimens was studied under static conditions, according to Russian National Standard GOST R 52126-2003 “Radioactive waste. Determination of chemical resistance”. Tests were performed in distilled water at room temperature (25–28 °C). Samples of the contact solution were taken 1, 3, 7, 10, 14, 21, and 28 days after the tests started. When testing irradiated ceramics, the non-irradiated sides of the specimens were covered with a waterproof varnish. Solution samples were analyzed for Mo and W content with inductively coupled plasma mass spectrometry using an ELEMENT™ 2 high resolution mass spectrometer (Thermo Scintific^®^, Bremen, Germany) with external calibration. Calibration was performed with ICP-MS-68A-B solution (High Purify Standards^®^, Charleston, SC, USA) using a Thermo Scientific^®^ ELEMENT^TM^ 2 high-resolution mass spectrometer (Thermo Scientific, Bremen, Germany).

**Figure 1:**
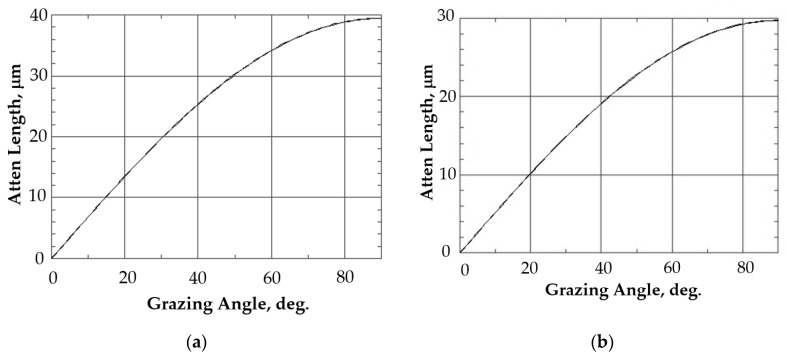
Calculating the depth of CuKα X-ray radiation penetration into the Na_0.5_Zr_2_(PO_4_)_2.5_(MoO_4_)_0.5_ (**a**) and Na_0.5_Zr_2_(PO_4_)_2.5_(WO_4_)_0.5_ (**b**) specimens depending on α incidence angle. Energy = 8000 eV.

In order to analyze the near-surface amorphous layer, a number of grazing incidence geometry (GIXRD) experiments were arranged. The GIXRD setup was equipped with a. parabolic Göbel mirror. With this geometric setup, the α angle between the specimen plane and the primary beam remained constant, while 2θ varied in the selected range of angles. In a series of experiments, α varied from 1° to 8° with an increment of 1°. Scanning in each experiment was carried out for the 2θ angle in the range from 22° to 24° using a point detector with an equatorial Soller slit. The depth of X-ray radiation penetration into the materials under study was calculated using a material X-ray properties database [78] and is shown in Figure 1. The α angle ranging from 1° to 8° corresponded to the penetration depth of 4–5 µm for the materials under study. The experiment focused on the dependence of integral intensity of diffraction peaks (211) and (031) for the Na_0.5_Zr_2_(PO_4_)_2.5_(WO_4_)_0.5_ phase and (113) for the Na_0.5_Zr_2_(PO_4_)_2.5_(MoO_4_)_0.5_ phase. The results were analyzed using the approach described earlier in [45,79].

The elemental composition of the ceramic surface layer was studied with secondary ion mass spectrometry (SIMS). Measurements were taken with the TOF.SIMS-5 setup, equipped with a time-of-flight mass analyzer with separate functions of probing and sputtering ion guns, operating in pulsed mode and not intersecting in time. A layer-by-layer analysis of the near-surface layer was carried out to a depth of about 500 nm with 25 keV Bi_3_^+^ cluster ions. Sputtering was carried out with 1 keV Cs^+^ ions. Measurements were taken in two modes of detecting secondary ions of both polarities (+ and −). Elementary and cluster secondary ions were detected in both modes.

The radiation stability of ceramics was assessed with high energy 167 MeV Xe^+26^ ion irradiation using an IC-100 FLNR JINR cyclotron (Joint Institute for Nuclear Research, Dubna, Russia). The specimens were irradiated at room temperature (23–27 °C) at fluences ranging from 1·10^12^ to 6·10^13^ cm^−2^. The average ion flux was about 2·10^9^ cm^−2^⋅s^−1^ to avoid any significant heating of targets. The temperature of targets during irradiation did not exceed 30 °C. Uniform distribution of the ion beam over the irradiated target surface was achieved with ion beam scanning. The accuracy of ion flux and fluence measurements reached 15%.

## 3. Results and Discussion

The ceramic specimens with high relative densities were obtained from Na-containing compounds by means of SPS. For research purposes, 10 ceramic specimens, with varying W content, and 10 specimens with varying Mo content were prepared. These specimens had no visible macro- and micro-cracks. They were produced in line with the modes specified in Part I. The average SPS time was 13 min for the Na_1−x_Zr_2_(PO_4_)_3−x_(MoO_4_)_x_ phosphate molybdates and 16 min for the Na_1−x_Zr_2_(PO_4_)_3−x_(WO_4_)_x_ phosphate tungstates. The ceramic specimen density was consistent with the data presented in Part 1. Densities close to the theoretical ones were ensured for almost all ceramics. Relative density of the ceramics with 0.4 and 0.5% Mo was 100.2–100.9% of theoretical density, while relative density of the ceramics with 0.4 and 0.5% W was 100.1–100.6% of theoretical value. We reckoned that the increased relative density of the ceramics stemmed from secondary phase impurities found in them. The results of XRD analysis presented in Part I indicated the presence of secondary phases in the ceramics. In W-containing ceramics, the Zr_2_(WO_4_)(PO_4_)_2_ secondary phase was identified, and in Mo-containing ceramics the CaCO_3_ and Al_3_O_0.34_Zr_5_ secondary phases were found.

**Figure 2:**
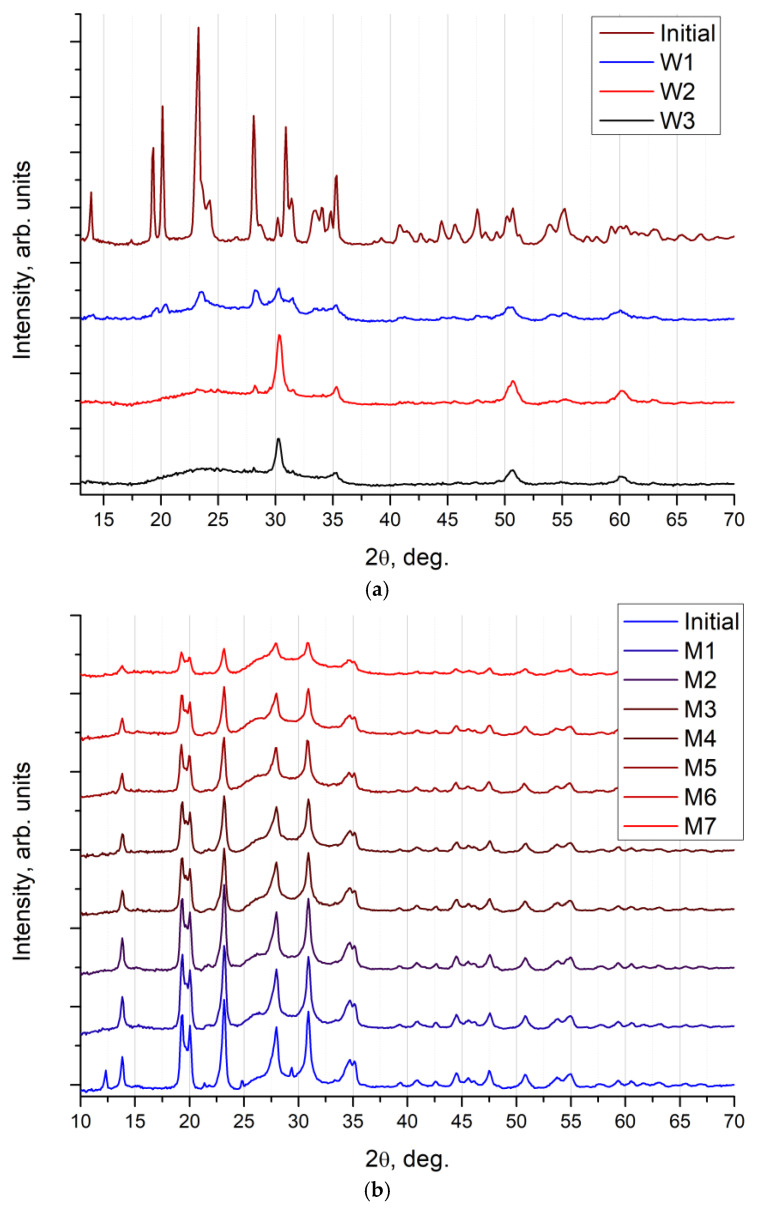
XRD curves of the phosphate tungstates specimens (**a**) and the phosphate molybdates specimens (**b**) with *x* = 0.5 after irradiation at the following fluences (cm^−2^). Initial state and when exposed to different Xe ion doses (in cm^−2^): (**a**): W1—3·10^12^; W2—10^13^; W3—3·10^13^; (**b**): M1—10^12^; M2 –3·10^12^; M3—6·10^12^; M4—8·10^12^; M5—10^13^; M6—3·10^13^; M7—6·10^13^.

Radiation stabilities of phosphate molybdates and phosphate tungstates were compared with the help of 167 MeV Xe ion irradiation at various fluences, ranging from 1·10^12^ to 6·10^13^ cm^–2^. The dose dependence of XRD curves registered in the Na_0.5_Zr_2_(PO_4_)_2.5_(XO_4_)_0.5_ ceramics is shown in Figure 2. The results of XRD analysis proved that the initial Na_0.5_Zr_2_(PO_4_)_2.5_(WO_4_)_0.5_ ceramics (Figure 2a) were amorphized when exposed to ion irradiation at a minimum dose of 3·10^12^ cm^−2^. Dose increase resulted in further amorphization and phase decomposition in Na_0.5_Zr_2_(PO_4_)_2.5_(WO_4_)_0.5_ accompanied by the ZrO_2_ phase formation. When exposed to 3·10^13^ cm^−2^ irradiation, no XRD peaks were observed in the Na_0.5_Zr_2_(PO_4_)_2.5_(WO_4_)_0.5_ on an XRD curve, only peaks in the crystalline ZrO_2_ phase and a wide halo of an amorphous component in the specimen remained.

As for the Na_0.5_Zr_2_(PO_4_)_2.5_(MoO_4_)_0.5_ ceramics (Figure 2b), XRD results showed a weak impact of ion irradiation on crystallinity of these ceramics. Diffraction peaks were seen clearly, even at irradiation doses of up to 6·10^13^ cm^−2^. Changes caused by ion irradiation in this series concerned the coherent scattering region sizes of the Na_0.5_Zr_2_(PO_4_)_2.5_(MoO_4_)_0.5_ phase slightly. However, ion irradiation did not result in critical degradation of crystallinity, as in the case of the Na_0.5_Zr_2_(PO_4_)_2.5_(WO_4_)_0.5_ ceramics. Intensity of XRD peaks reduced 4 times with an increase in the irradiation dose of the Na_0.5_Zr_2_(PO_4_)_2.5_(MoO_4_)_0.5_ ceramics (Figure 2b).

**Figure 3:**
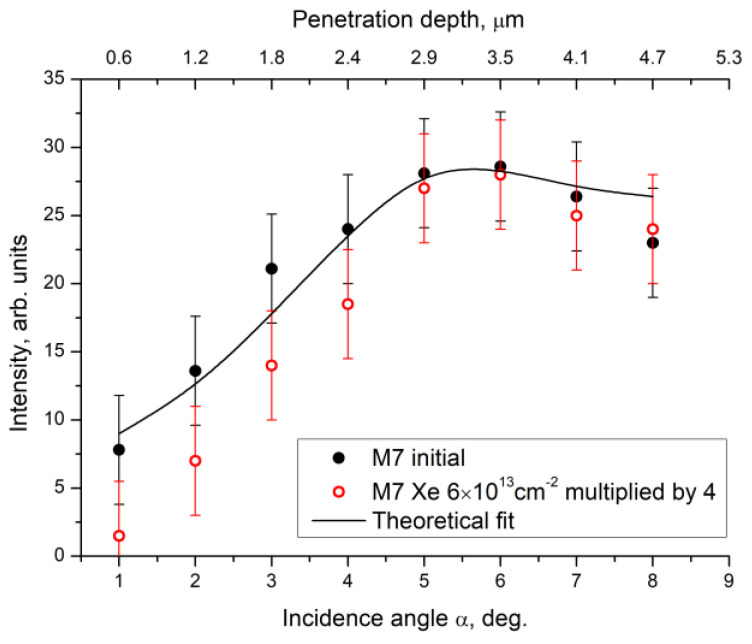
Dependence of integral intensity of a XRD peak (113) in Na_0.5_Zr_2_(PO_4_)_2.5_(MoO_4_)_0.5_ phase on α incidence angle for Na_0.5_Zr_2_(PO_4_)_2.5_(MoO_4_)_0.5_ ceramics before and after irradiation. Irradiation intensity is multiplied by a factor of 4. The calculated curve is plotted with due regard for material constants and the geometry of the experiment as if crystalline quality and phase composition of the material were uniform over the entire depth of analysis.

Figure 3 shows the dependence of the intensity of an XRD peak (113) on X-ray incidence angle in Na_0.5_Zr_2_(PO_4_)_2.5_(MoO_4_)_0.5_ ceramics in the initial state and after irradiation at a dose of 6·10^13^ cm^−2^. The intensity for all experimental points of the irradiated specimen was multiplied by a factor of 4 for easier data comparison. Figure 3 shows a calculated curve plotted with due regard for material constants, and the geometry of the experiment as if crystalline quality and phase composition of the material were uniform over the entire depth of analysis. It was apparent that dependences were the same for the initial and irradiated specimens. It could be assumed that, within the depth of analysis of 5 µm, the degree of amorphization of the near-surface layer was the same and approximated 75% for M7 ceramics (irradiation dose of 6·10^13^ cm^−2^).

**Figure 4:**
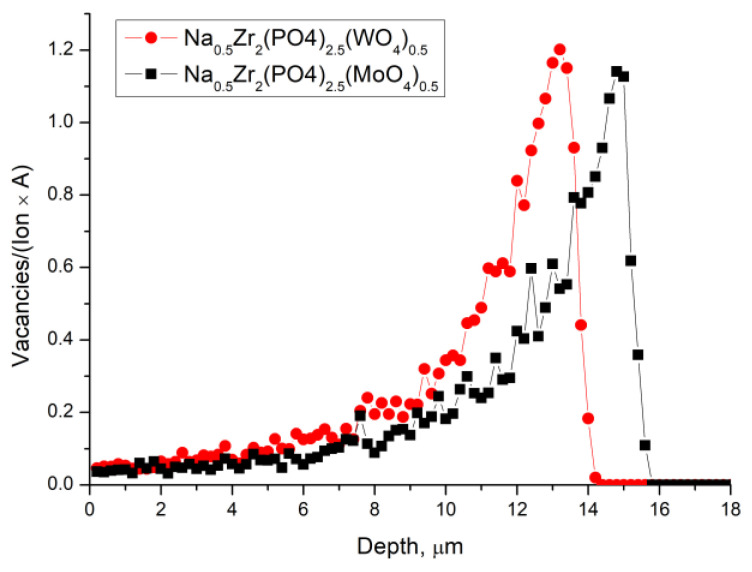
Results of SRIM simulation of vacancies depth distribution caused by 167 MeV Xe ions in M- and W-series specimens.

To assess how thick the damaged layer was, the depth of Xe ions penetration into the surface layers of the materials was simulated using SRIM-2013 software [80], in accordance with the method proposed in [81]. The results of simulating the distribution of vacancies depth for both materials under study are shown in Figure 4. It was apparent that the depth of the damaged layer significantly exceeded the depth of analysis in the GIXRD method for these materials and ion beam parameters. The assumption about uniform amorphization of the near-surface layer, about 5 μm thick, was confirmed by the simulation results. It could be seen that, in W-containing ceramics, the depth of defect formation was somewhat less, and the concentration of defects in the near-surface layer was slightly greater. We could assume that the energy of the ions was more efficiently transferred to the W-containing material, which led to its amorphization at lower doses. Probable explanations might involve differences in W and Mo chemical bonds with the environment and also in the atomic mass of W and Mo. This assumption agreed with the results of XRD analysis in the symmetric Bragg–Brentano geometry and was previously observed in other W- and Mo-containing ceramics [79].

**Figure 5:**
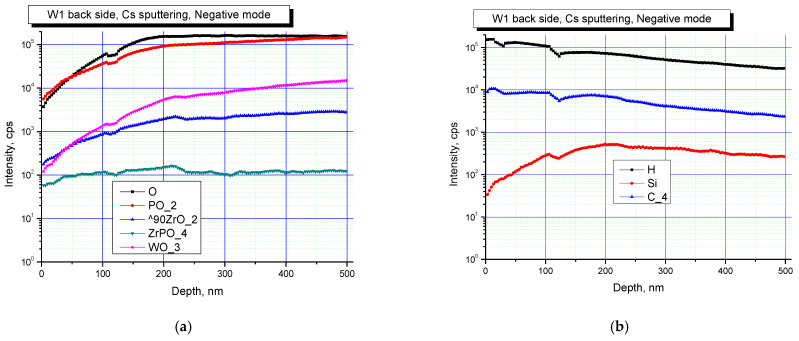
Profiles of main (**a**) and doping (**b**) elements on the surface of the Na_0.5_Zr_2_(PO_4_)_2.5_(WO_4_)_0.5_ ceramics in negative secondary ion detection mode.

**Figure 6:**
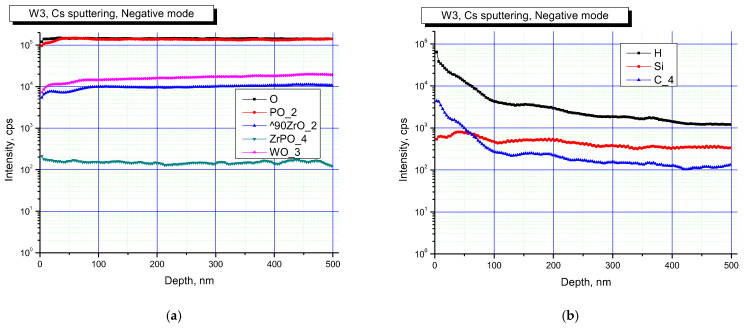
Profiles of main (**a**) and doping (**b**) elements on the surface of the irradiated Na_0.5_Zr_2_(PO_4_)_2.5_(WO_4_)_0.5_ ceramics in negative secondary ion detection mode.

Figure 5 and Figure 6 show the results of SIMS studies of the surface layers of Na_0.5_Zr_2_(PO_4_)_2.5_(WO_4_)_0.5_ ceramics. Concentration depth profiles for the specimens in the initial state, and when exposed to irradiation at a dose of 3·10^13^ cm^−2^, are shown in negative secondary ion detection mode.

The analysis of results presented in Figure 5 and Figure 6 allows for a conclusion that the surface of all the specimens contained P, Zr, W oxides. The studies also indicated that the surface of the specimens was partially contaminated with Si. Irradiation led to increased contributions of P and Zr oxides. The changes observed might stem from changes in the phase composition of the specimens after irradiation, which led to a change in the probabilities of formation and the release of various cluster secondary ions.

It is noteworthy that the surface layer contained P, Zr, and W oxides, as well as a high concentration of C. Contamination of ceramics surface layers during SPS is one of the known drawbacks of this method, which has been described in a variety of research articles [45,82,83,84,85,86,87,88,89,90,91]. Carburization of surface layers during SPS occurs because the sintered material interacts with a graphite dye or graphite foil used to improve contact between the specimen surface and the inner wall of a graphite dye.

**Figure 7:**
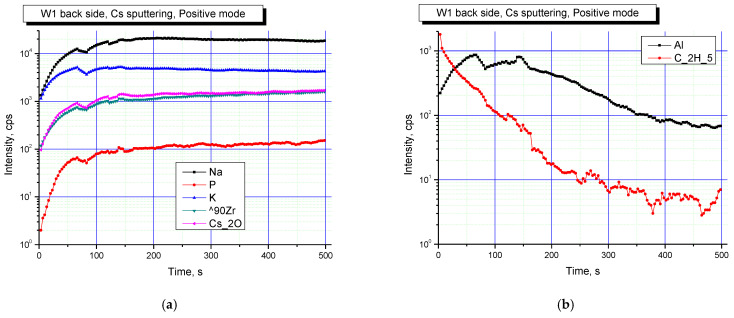
Profiles of main (**a**) and doping (**b**) elements on the surface of the Na_0.5_Zr_2_(PO_4_)_2.5_(WO_4_)_0.5_ ceramics in the initial state. Positive secondary ion detection mode. SIMS.

**Figure 8:**
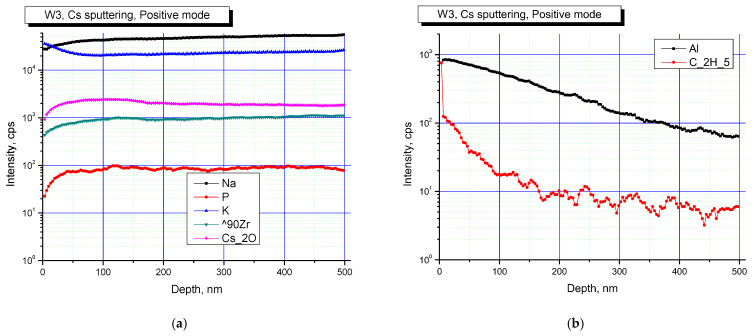
Profiles of main (**a**) and doping (**b**) elements on the surface of the irradiated Na_0.5_Zr_2_(PO_4_)_2.5_(WO_4_)_0.5_ ceramics. Positive secondary ion detection mode. SIMS.

Figure 7 and Figure 8 show elemental profiles of the initial and irradiated Na_0.5_Zr_2_(PO_4_)_2.5_(WO_4_)_0.5_ ceramic specimens in positive secondary ion detection mode.

Figure 7 and Figure 8 show the results of studies in positive secondary ion detection mode. These results suggested that Na, K, Zr were present in all the specimens, and after irradiation, Na and K contributions increased. Doping elements or impurities in the specimens were mostly Al and hydrocarbon contaminants (C_2_H_5_ cluster line). After irradiation, the contribution of hydrocarbons decreased.

The hydrolytic stabilities of the specimens with high Mo and W contents (x = 0.4 and 0.5) were studied. According to XRD data, no crystal structure damage was observed during the hydrolytic tests. Unit cell parameters of Na_1–x_Zr_2_(PO_4_)_3–x_(XO_4_)_x_ were identical before and after the hydrolytic tests. Normalized release rates per unit surface area (*R*) for particular components were determined according to the formulae:

*R* = *NL*/*t*, (1)

*NL* = *m*/(*ω*·*S*), (2)

where *m* [g] is the mass of a component leached for a given time, *t* [days] is the test duration, *S* [cm^2^] is the open surface area, and *ω* is the mass fraction of the component in the initial specimen.

**Figure 9:**
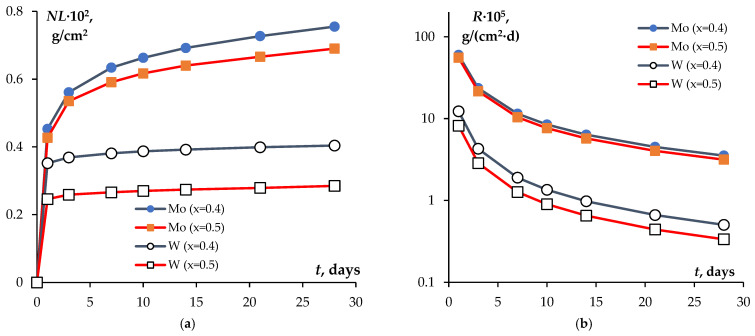
Time dependences of normalized weight loss *NL* (**a**) and normalized leaching rates for certain components per unit surface area *R* (**b**) in the Na_1−x_Zr_2_(PO_4_)_3−x_(XO_4_)_x_ ceramics.

The normalized weight loss values *NL* and the normalized leaching rates *R* after 28 days of testing are presented in Table 1. Time dependencies of the above values are shown in Figure 9. The normalized leaching rates after 28 days of testing (*R*_min_) were 31.6·10^−6^ g·cm^−2^·day^−1^ for Mo-containing compounds (*x* = 0.5) and 3.36·10^−6^ g·cm^−2^·day^−1^ for W-containing ones (*x* = 0.5). After 28 days of testing, the normalized leaching rate for Mo from the Na_1−x_Zr_2_(PO_4_)_3−x_(XO_4_)_x_ ceramics at x = 0.4 and 0.5 was by an order of magnitude greater than that of W. This was a very low normalized leaching rate after 28 days of testing of NZP ceramics suggesting their having high chemical resistance. We reckoned that this result indicated that inorganic compounds of the NZP family could have advanced applications as binders for W- and Mo-containing fractions of HLW.

As follows from Table 1 and Figure 9, the normalized leaching rates *R* decreased while W and Mo contents increased. At the moment, we have no clear explanation of this effect. In our opinion, it might be specific to a stationary mode of testing phosphate tungstates and phosphate molybdates i.e., metal atoms that are leached react with oxygen diluted in water, which results in thin oxide films that form on ceramics surfaces and prevent further leaching of heavy metals. The surface area covered with an oxide film grows along with an increase in W and/or Mo contents in ceramics.

**Figure 10:**
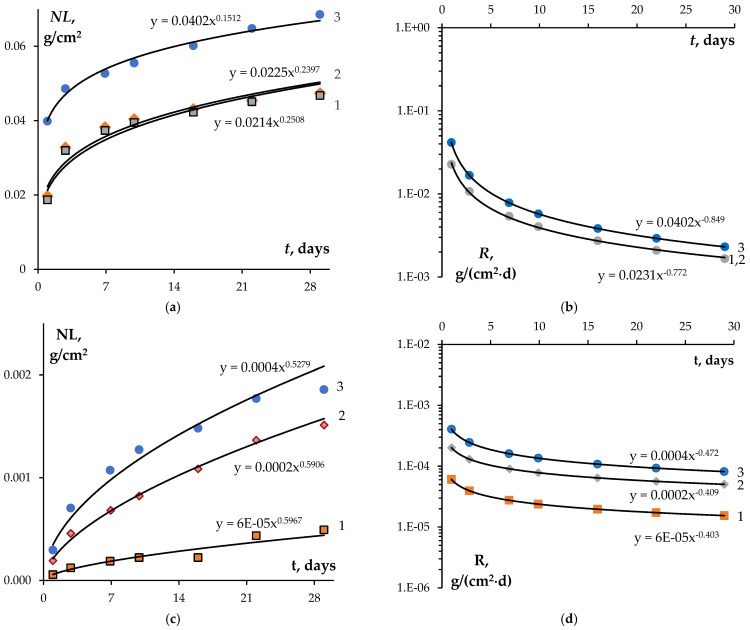
Dependence of normalized weight loss (*NL*) (**a**,**c**) and leaching rate (*R*) (**b**,**d**) on testing time *t* for the Na_0.5_Zr_2_(PO_4_)_2.5_(MoO_4_)_0.5_ (**a**,**b**) and Na_0.5_Zr_2_(PO_4_)_2.5_(WO_4_)_0.5_ (**c**,**d**) ceramic specimens. Fluence: 1–3·10^12^ cm^−2^, 2–1·10^13^ cm^−2^, 3–3·10^13^ cm^−2^.

Figure 10 shows the results of hydrolytic testing of the Na_0.5_Zr_2_(PO_4_)_2.5_(MoO_4_)_0.5_ (a, b) and Na_0.5_Zr_2_(PO_4_)_2.5_(WO_4_)_0.5_ ceramics after irradiation at different doses. *NL(t)* and *R(t)* data were interpolated with a power function. The analysis of *R_i_(t)* and *NL_i_(t)* dependencies showed that higher doses increased W and Mo leaching rates. The Mo leaching rate turned out to be significantly higher than that of W. After irradiation at a fluence of 3·10^12^ cm^−2^, the Mo leaching rate after 28 days of testing was *R_Mo_* = 1.6·10^−3^ g⋅cm^−2^⋅d^−1^, while the W leaching rate was *R_W_* = 8.2·10^−5^ g⋅cm^−2^⋅d^−1^. After irradiation at a dose of 3·10^13^ cm^−2^, the Mo leaching rate increased to 2.3·10^−3^ g⋅cm^−2^⋅d^−1^, while the W leaching rate reached 5.0·10^−5^ g⋅cm^−2^⋅d^−1^. Comparison of these results with the data presented in Table 1 shows that the hydrolytic stability of the irradiated ceramics decreased, but remained high for the W-containing ceramics.

Let us compare the leaching rate *R* estimates with literature data. It was noted that key data on resistance of NZP ceramics obtained by conventional methods are presented in many works (see, for example, [2,6,92,93,94,95,96,97,98], etc.). Here we shall point out only some data that is crucial in order to analyze the results obtained. NZP ceramics demonstrated high chemical resistance, including after irradiation. It is known that NZP ceramics do not decompose even after 2 years of exposure to hydrothermal conditions at 400 °C, including after irradiation with ^60^Co [99,100]. According to [101], the Ca leaching rate *R* for Ca_0.75_Zr_2_(PO_4_)_2.5_(SiO_4_)_0.5_, obtained by cold pressing (P = 200 MPa) followed by sintering (900 °C, 10 h), was ~1·10^-8^ g⋅cm^−2^⋅day^−1^. Tests were carried out under static conditions at room temperature for 21 days. As shown in [92], the Zr leaching rate for La_1/3_Zr_2_(PO_4_)_3_ under static conditions was less than 10^−5^ g⋅m^−2^⋅day^−1^, while the La leaching rate depended on the ratio of the ceramic surface area to the solution volume and was ~10^−6^ g⋅m^−2^⋅day^−1^ after 14 days of testing. According to [102], the Pu leaching rate in Pu_1/3_Zr_2_(PO_4_)_3_ after testing for 14 days at room temperature under static conditions was ~9.9·10^−6^ g⋅cm^−2^⋅day^−1^. Pu_1/3_Zr_2_(PO_4_)_3_ ceramics was produced by cold pressing of powders (P = 200 MPa) and sintering at 950 °C (7 h). Low Sr leaching rates (less than 10^−6^ g⋅m^−2^⋅day^−1^) at room temperature in deionized water were measured for ceramics obtained by thermal treatment of HZr_2_(PO_4_)_3_ + Sr(NO_3_)_2_ [103]. The same high chemical stability of NZP ceramics was found during tests based on the Soxhlet method [104], as well as during tests of multicomponent compounds with an NZP structure that simulated the composition of various RAW fractions [105]. The Cs leaching rate in CsMgPO_4_, CsZr_2_(PO_4_)_3_, and Cs_2_Mg_0.5_Zr_1.5_(PO_4_)_3_ specimens obtained by SPS varied between 3·10^−4^ and 4·10^−6^ g⋅m^−2^⋅day^−1^ [106,107]. As shown in [44], in NaRe_2_(PO_4_)_3_ ceramics with relative density of 85% obtained by SPS under static conditions at room temperature, the Re leaching rate was 1.3·10^−5^ g⋅cm^−1^⋅day^−1^. Higher Re leaching rates were explained in [44] by the low density of ceramics and, as a result, the large specific surface area. Given high leaching rates of Mo and W from the structure of phosphates (see, for example, [108]), it could be assumed that the ceramics obtained had high chemical stability.

**Figure 11:**
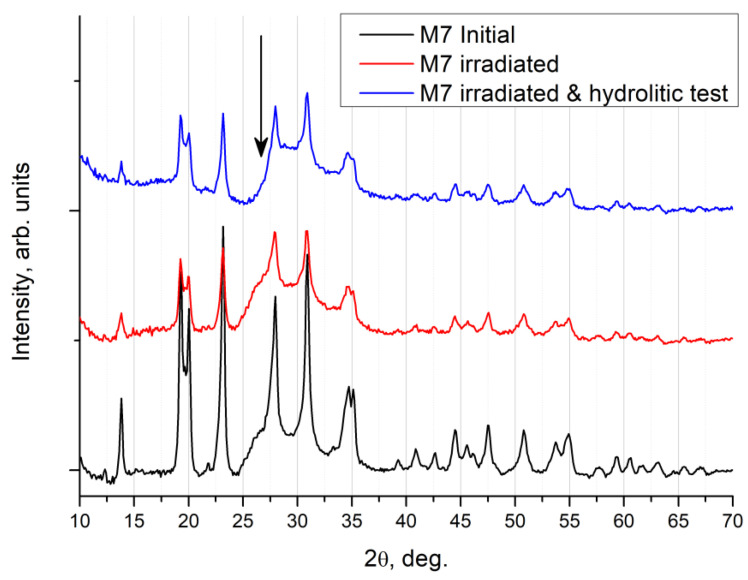
XRD patterns for the Na_0.5_Zr_2_(PO_4_)_2.5_(MoO_4_)_0.5_ ceramic specimen: initial state, irradiated with Xe ions at a dose of 6·10^13^ cm^−2^, and after hydrolytic tests.

Figure 11 shows the XRD results in the symmetric Bragg–Brentano geometry of the surface of the irradiated Na_0.5_Zr_2_(PO_4_)_2.5_(MoO_4_)_0.5_ ceramics after the hydrolytic tests.

The Na_0.5_Zr_2_(PO_4_)_2.5_(MoO_4_)_0.5_ ceramics after hydrolytic tests showed no change in peak intensity of the main phase, and no peaks of auxiliary phases could be observed. A broad peak of a microcrystalline phase in graphite (shown by an arrow in the figure near 26° in 2θ) disappeared, which was the only change. Apparently, a side phase of graphite was washed out from the near-surface layer and from the pores of the specimen as a result of the hydrolytic tests.

**Figure 12:**
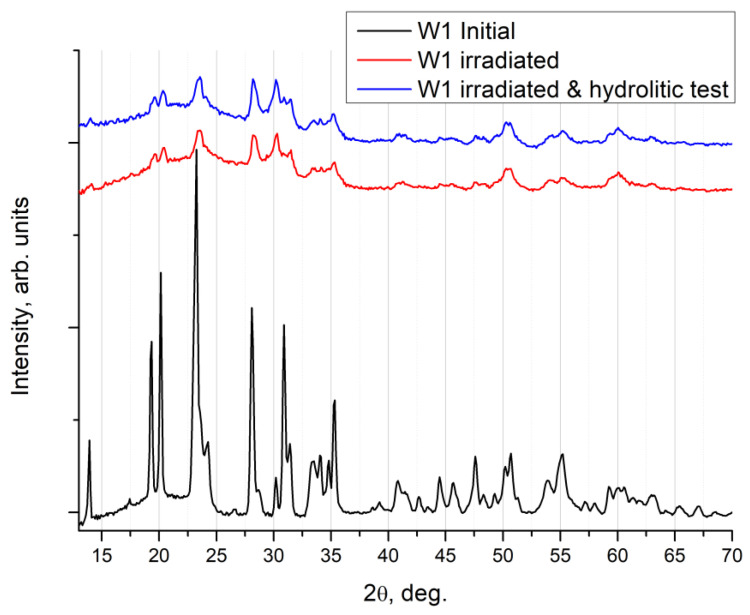
XRD patterns for the Na_0.5_Zr_2_(PO_4_)_2.5_(WO_4_)_0.5_ ceramic specimen: initial state, irradiated with Xe ions at a dose of 3·10^12^ cm^−2^, and after hydrolytic tests.

Representative data of an XRD experiment in the symmetric Bragg–Brentano geometry for the Na_0.5_Zr_2_(PO_4_)_2.5_(WO_4_)_0.5_ ceramics is shown in Figure 12.

With the W-containing ceramics, it was apparent that the hydrolytic tests did not lead to a change in peak intensity of the main phase in Na_0.5_Zr_2_(PO_4_)_2.5_(WO_4_)_0.5_, and no peaks of auxiliary phases could be observed. There are no intensity changes near 26° associated with the graphite phase. Apparently, a graphite phase was initially absent in the W-series specimens since they were less porous or had other preparation-induced features. Carbon contamination, detected with SIMS, stemmed from an increase in the content of carbon ions in the Na_0.5_Zr_2_(PO_4_)_2.5_(WO_4_)_0.5_ crystal lattice. As noted earlier, this might be due to the intense diffusion of carbon from the graphite mold or graphite paper, with which the specimen surface interacted during SPS.

## 4. Conclusions

XRD analysis showed that the structure of the compounds Na_1−x_Zr_2_(PO_4_)_3−x_(XO_4_)_x_ remained unchanged during sintering and hydrolytic stability tests. The normalized leaching rates after 28 days of testing were 31·10^−6^ g·cm^−2^·d^−1^ for compounds Na_0.5_Zr_2_(PO_4_)_2.5_(MoO_4_)_0.5_ and 3.36·10^−6^ g·cm^−2^·d^−1^ for Na_0.5_Zr_2_(PO_4_)_2.5_(WO_4_)_0.5_ ones.

Irradiation tests proved that the destruction of the NZP crystal lattice was less expressed in the Mo-containing specimens, as compared to phosphate tungstates irradiated under similar conditions. The crystal lattice of W-containing ceramic specimens broke down as a result of irradiation at a fluence of 3·10^13^ cm^−2^.

Irradiation led to an increase in the leaching rate of W and Mo from the crystal structure of the ceramics. The irradiated W-containing ceramics had higher hydrolytic resistance, compared to the Mo-containing NZP ceramics. The leaching rates observed on the 28th day of testing for the irradiated Na_0.5_Zr_2_(PO_4_)_2.5_(MoO_4_)_0.5_ specimens were 1.6⋅10^−3^ g⋅cm^−2^⋅d^−1^ at a fluence of 3·10^12^ cm^−2^ and 2.3·10^−3^ g⋅cm^−2^⋅d^−1^ at a fluence of 3·10^13^ cm^−2^. The Na_0.5_Zr_2_(PO_4_)_2.5_(WO_4_)_0.5_ ceramics after irradiation at similar fluences had the leaching rates of 8.2·10^−5^ and 5.0·10^−5^ g⋅cm^−2^⋅d^−1^, respectively.

## Figures and Tables

**Table 1:** Normalized weight loss (*NL*) and normalized leaching rates (*R*) after 28 days of testing for Mo and W in the Na_1−x_Zr_2_(PO_4_)_3−x_(XO_4_)_x_ ceramics.

x	*t*, Days	*m*·10^4^, g		*NL*·10^2^, g·cm^−2^		*R*·10^5^, g·cm^−2^·d^−1^	
		Mo	W	Mo	W	Mo	W
0.4	1	5.917	8.333	0.453	0.352	60.000	12.300
	3	1.417	0.417	0.561	0.369	23.583	4.289
	7	0.958	0.275	0.634	0.381	11.477	1.903
	10	0.375	0.133	0.663	0.387	8.475	1.352
	14	0.375	0.125	0.692	0.392	6.367	0.979
	21	0.458	0.167	0.727	0.399	4.511	0.664
	28	0.375	0.125	0.755	0.404	3.532	0.504
0.5	1	7.250	7.500	0.427	0.246	55.600	8.200
	3	1.833	0.392	0.535	0.259	21.591	2.859
	7	0.958	0.208	0.591	0.266	10.410	1.269
	10	0.433	0.125	0.617	0.270	7.657	0.901
	14	0.400	0.108	0.640	0.274	5.731	0.653
	21	0.442	0.167	0.666	0.279	4.042	0.442
	28	0.408	0.167	0.690	0.285	3.156	0.336

## Data Availability

Not applicable.

## References

[1] Orlova A.I., Ojovan M.I. (2019). Ceramic mineral waste-forms for nuclear waste immobilization. Materials.

[2] Bohre A., Avasthi K., Pet’kov V.I. (2017). Vitreous and crystalline phosphate high level waste matrices: Present status and future challenges. J. Ind. Eng. Chem..

[3] Ewing R., Wang L. (2002). Phosphates as Nuclear Waste forms. Review in Mineralogy and Geochemistry. Phosphates Geochem. Geobiol. Mater. Importance.

[4] Orlova A.I. (2022). Crystalline phosphates for HLW immobilization—Composition, structure, properties and production of ceramics. Spark Plasma Sintering as a promising sintering technology. J. Nucl. Mater..

[5] Savinykh D.O., Khainakov S.A., Orlova A.I., Garcia-Granda S. (2018). New phosphate-sulfates with NZP Structure. Russ. J. Inorg. Chem..

[6] Pet’kov V., Asabina E., Loshkarev V., Sukhanov M. (2016). Systematic investigation of the strontium zirconium phosphate ceramic form for nuclear waste immobilization. J. Nucl. Mater..

[7] Scheetz B.E., Agrawal D.K., Breval E., Roy R. (1994). Sodium zirconium phosphate (NZP) as a host structure for nuclear waste immobilization: A review. Waste Manag..

[8] Scales N., Dayal P., Aughterson R.D., Zhang Y., Gregg D.I. (2022). Sodium zirconium phosphate-based glass-ceramics as potential wasteforms for the immobilization of nuclear wastes. J. Am. Cer. Soc..

[9] Ojovan M.I., Lee W.E., Kalmykov S.N. (2019). An Introduction to Nuclear Waste Immobilisation.

[10] Weber W.J., Navrotsky A., Stefanovsky S., Vance E.R., Vernaz E. (2009). Materials Science of High-Level Nuclear Waste Immobilization. MRS Bull..

[11] Ewing R.C. (2001). The design and evaluation of nuclear-waste forms: Clues from mineralogy. Can. Mineral..

[12] Lumpkin G.R. (2006). Ceramic waste forms for actinides. Elements.

[13] Terra O., Dacheux N., Audubert F., Podor R. (2006). Immobilization of tetravalent actinides in phosphate ceramics. J Nucl. Mater..

[14] Dacheux N., Clavier N., Podor R. (2013). Versatile Monazite: Resolving geological records and solving challenges in materials science. Monazite as a promising long-term radioactive waste matrix: Benefits of high-structural flexibility and chemical durability. Am. Mineral..

[15] Ananthanarayanan A., Ambashta R.D., Sudarsan V., Ajithkumar T., Sen D., Mazumber S., Wattal P.K. (2017). Structure and short time degradation studies of sodium zirconium phosphate ceramics loaded with simulated fast breeder (FBR) waste. J. Nucl. Mater..

[16] Pet’kov V.I., Sukhanov M.V., Kurazhkovskaya V.S. (2003). Molybdenum fixation in crystalline NZP matrices. Radiochemistry.

[17] Chourasiaa R., Shrivastavaa O.P., Wattal P.K. (2009). Synthesis, characterization and structure refinement of sodium zirconium molibdato-phosphate: Na_0.9_Zr_2_Mo_0.1_P_2.9_O_12_ (MoNZP). J. Alloys Compd..

[18] Bohre A., Shrivastava O.P., Avasthi K. (2016). Solid state synthesis and structural refinement of polycrystalline phases: Ca_1−2x_Zr_4_M_2x_P_6−2x_O_24_ (M = Mo, x = 0.1 and 0.3). Arab. J. Chem..

[19] Kumar S.P., Gopal B. (2011). Immobilization of “Mo^6+^” in monazite lattice: Synthesis and characterization of new phosphomolybdates, La_1−x_Ca_x_P_1−y_Mo_y_O_4_, where x = y = 0.1–0.9. J. Am. Cer. Soc..

[20] Daub M., Lehner A.J., Höppe H.A. (2012). Synthesis, crystal structure and optical properties of Na_2_RE(PO_4_)(WO)_4_ (RE = Y,Tb-Lu). Dalton Trans..

[21] Sukhanov M.V., Pet’kov V.I., Kurazhkovskaya V.S., Eremin N.N., Urusov V.S. (2006). Computer-assisted structure simulation, synthesis, and phase formation of molybdophosphates A_1−x_Zr_2_(PO_4_)_3−x_(MoO_4_)_x_ (A is an alkali metal). Russ. J. Inorg. Chem..

[22] Bennouna L., Arsalane S., Brochu R., Lee M.R., Chassaing J., Quarton M. (1995). Spécificités des ions Nb^IV^ and Mo^IV^ dans les monophosphates de type Nasicon. J. Solid State Chem..

[23] Buzlukov A.L., Fedorov D.S., Serdtsev A.V., Kotova I.Y., Tyutyunnik A.P., Korona D.V., Baklanova Y.V., Ogloblichev V.V., Kozhevnikova N.M., Denisova T.A. (2022). Ion mobility in triple sodium molybdates and tungstates with a NASICON structure. J. Exp. Theor. Phys..

[24] Vereshchagina T.A., Fomenko E.V., Vasilieva N.G., Solovyov L.A., Vereshchagin S.N., Bazarova Z.G., Anshits A.G. (2011). A novel layered zirconium molybdate as a precursor to a ceramic zirconomolybdate host for lanthanide bearing radioactive waste. J. Mater. Chem..

[25] Tokita M. (2021). Progress of Spark Plasma Sintering (SPS): Method, Systems, Ceramics Applications and Industrialization. Ceramics.

[26] Olevsky E., Dudina D. (2018). Field-Assisted Sintering: Science and Applications.

[27] Dudina D.V., Vidyuk T.M., Korchagin M.A. (2021). Synthesis of ceramic reinforcements in metallic matrices during Spark Plasma Sintering: Consideration of Reactant/Matrix Mutual Chemistry. Ceramics.

[28] Hu Z.-Z., Zhang Z.-H., Cheng X.-W., Wang F.-C., Zhang Y.-F., Li S.-L. (2020). A review of multi-physical fields induced phenomena and effects in spark plasma sintering: Fundamentals and applications. Mater. Des..

[29] Grasso S., Sakka Y., Maizza G. (2009). Electric current activated/assisted sintering (ECAS): A review of patents 1906–2008. Sci. Technol. Adv. Mater..

[30] Wang L., Zhang J., Jiang W. (2013). Recent development in reactive synthesis of nanostructured bulk materials by spark plasma sintering. Int. J. Refract. Met. Hard Mater..

[31] Yamanoglu R. (2019). Pressureless Spark Plasma Sintering: A perspective from conventional sintering to accelerated sintering without pressure. Powder Metall. Met. Ceram..

[32] Kai R., Mukhopadhyay A. (2014). Spark plasma sintered/synthesized dense and nanostructured materials for solid-state Li-ion batteries: Overview and perspective. J. Power Source.

[33] Chaim R., Chevallier G., Weibel A., Estournès C. (2018). Grain growth during spark plasma and flash sintering of ceramic nanoparticles: A review. J. Mater. Sci..

[34] Dudina D.V., Bokhonov B.B., Olevsky E.A. (2019). Fabrication of porous materials by spark plasma sintering: A review. Materials.

[35] Hulbert D.M., Anders A., Dudina D.V., Andersson J., Jiang D., Unuvar C., Anselmi-Tamburini U., Lavernia E.J., Mukherjee A.K. (2008). The absence of plasma in “spark plasma sintering”. J. Appl. Phys..

[36] Mukasyan A.S., Rogachev A.S., Moskovskikh D.O., Yermekova Z.S. (2022). Reactive spark plasma sintering of exothermic systems: A critical review. Ceram. Int..

[37] Munir Z.A., Anselmi-Tamburini U., Ohyanagi M. (2006). The effect of electric field and pressure on the synthesis and consolidation of materials: A review of the spark plasma sintering method. J. Mater. Sci..

[38] Sukhanov M.V., Pet’kov V.I., Firsov D.V. (2011). Sintering mechanism for high-density NZP ceramics. Inorg. Mater..

[39] Shichalin O.O., Papynov E.K., Maiorov V.Y., Belov A.A., Modin E.B., Buravlev I.Y., Azarova Y.A., Golub A.V., Gridasova E.A., Sukhorada A.E. (2019). Spark Plasma Sintering of Aluminosilicate Ceramic Matrices for Immobilization of Cesium Radionuclides. Radiochemistry.

[40] Papynov E.K., Belov A.A., Shichalin O.O., Buravlev I.Y., Azon S.A., Gridasova E.A., Parotkina Y.A., Yagofarov V.Y., Drankov A.N., Golub A.V. (2021). Synthesis of perovskite-like SrTiO_3_ ceramics for radioactive strontium immobilization by spark plasma sintering-reactive synthesis. Russ. J. Inorg. Chem..

[41] Shichalin O.O., Papynov E.K., Nepomnyushchaya V.A., Ivanets A.I., Belov A.A., Dran’kov A.N., Yarusova S.B., Buravlev I.Y., Tarabanova A.E., Fedorets A.N. (2022). Hydrothermal synthesis and spark plasma sintering of NaY zeolite as solid-state matrices for cesium-137 immobilization. J. Eur. Ceram. Soc..

[42] Orlova A.I., Troshin A.N., Mikhailov D.A., Chuvil’deev V.N., Boldin M.S., Sakharov N.V., Nokhrin A.V., Skuratov V.A., Kirilkin N.S. (2014). Phosphorous-containing cesium compounds of pollucite structure. Preparation of high-density ceramic and its radiation test. Radiochemistry.

[43] Potanina E., Golovkina L., Orlova A., Nokhrin A., Boldin M., Sakharov N. (2016). Lanthanide (Nd, Gd) compounds with garnet and monazite structures. Powders synthesis by "wet" chemistry to sintering ceramics by Spark Plasma Sintering. J. Nucl. Mater..

[44] Alekseeva L.S., Nokhrin A.V., Orlova A.I., Boldin M.S., Lantsev E.A., Murashov A.A., Korchenkin K.K., Ryabkov D.V., Chuvil’deev V.N. (2022). Ceramics based on the NaRe_2_(PO_4_)_3_ phosphate with the kosnarite structure as waste forms for technetium immobilization. Inorg. Mater..

[45] Mikhaylov D.A., Potanina E.A., Nokhrin A.V., Orlova A.I., Yunin P.A., Sakharov N.V., Boldin M.S., Belkin O.A., Skuratov V.A., Issatov A.T. (2022). Investigation of the Microstructure of Fine-Grained YPO_4_:Gd Ceramics with Xenotime Structure after Xe Irradiation. Ceramics.

[46] Alekseeva L., Nokhrin A., Boldin M., Lantsev E., Murashov A., Orlova A., Chuvil’deev V. (2021). Study of the hydrolytic stability of fine-grained ceramics based on Y_2.5_Nd_0.5_AlO_12_ oxide with a garnet structure under hydrothermal conditions. Materials.

[47] Papynov E.K., Shichalin O.O., Mayorov V.Y., Kuryavyi V.G., Kaidalova T.A., Teplukhina L.V., Portnyagin A.S., Slobodyuk A.B., Belov A.A., Tananaev I.G. (2019). SPS technique for ionizing radiation source fabrication based on dense cesium-containing core. J. Hazardous Mater..

[48] Mikhailov D.A., Orlova A.I., Malanina N.V., Nokhrin A.V., Potanina E.A., Chuvil’deev V.N., Boldin M.S., Sakharov N.V., Belkin O.A., Kalenova M.Y. (2018). A study of fine-grained ceramics based on complex oxides ZrO_2_-Ln_2_O_3_ (Ln = Sm, Yb) obtained by Spark Plasma Sintering for inert matrix fuel. Ceram. Int..

[49] Orlova A.I., Volgutov V.Y., Mikhailov D.A., Bykov D.M., Skuratov V.A., Chuvil’Deev V.N., Nokhrin A.V., Boldin M.S., Sakharov N.V. (2014). Phosphate Ca_1/4_Sr_1/4_Zr_2_(PO_4_)_3_ of the NaZr_2_(PO_4_)_3_ structure type: Synthesis of a dense ceramic material and its radiation testing. J. Nucl. Mater..

[50] Shichalin O.O., Yarusova S.B., Ivanets A.I., Papynov E.K., Belov A.A., Azon S.A., Buravlev I.Y., Panasenko A.E., Zadorozhny P.A., Mayorov V.Y. (2022). Synthesis and spark plasma sintering of solid-state matrices based on calcium silicate for ^60^Co immobilization. J. Alloys Compd..

[51] Shichalin O.O., Belov A.A., Zavyalov A.P., Papynov E.K., Azon S.A., Fedorets A.N., Buravlev I.Y., Balanov M.I., Tananaev I.G., Shi Y. (2022). Reaction synthesis of SrTiO_3_ mineral-like ceramics for strontium-90 immobilization via additional in situ synchrotron studies. Ceram. Int..

[52] Papynov E.K., Shichalin O.O., Buravlev I.Y., Belov A.A., Portnyagin A.S., Fedorets A.N., Azarova Y.A., Tananaev I.G., Sergienko V.I. (2020). Spark plasma sintering-reactive synthesis of SrWO_4_ ceramic matrices for ^90^Sr immobilization. Vacuum.

[53] Ge L., Subhash G., Baney R.H., Tulenko J.S., McKenna E. (2013). Densification of uranium dioxide fuel pellets prepared by spark plasma sintering (SPS). J. Nucl. Mater..

[54] Cologna M., Tyrpekl V., Ernstberger M., Stohr S., Somers J. (2016). Sub-micrometre grained UO_2_ pellets consolidated from sol gel beads using spark plasma sintering (SPS). Ceram. Int..

[55] Papynov E.K., Shichalin O.O., Mironenko A.Y., Ryakov A.V., Manakov I.V., Makhrov P.V., Buravlev I.Y., Tananaev I.G., Avramenko V.A., Sergienko V.I. (2018). Synthesis of high-density pellets of uranium dioxide by spark plasma sintering in dies of different types. Radiochemistry.

[56] Malkki P., Jolkkonen M., Hollmer T., Wallenius J. (2014). Manufacture of fully dense uranium nitride pellets using hydride derived powders with spark plasma sintering. J. Nucl. Mater..

[57] Johnson K.D., Wallenius J., Jolkkonen M., Claisse A. (2016). Spark plasma sintering and porosity studies of uranium nitride. J. Nucl. Mater..

[58] Yang K., Kardoulaki E., Zhao D., Broussard A., Metzger K., White J.T., Sivack M.R., Mcclellan K.J., Lahoda E.J., Lian J. (2021). Uranium nitride (UN) pellets with controllable microstructure and phase—Fabrication by spark plasma sintering and their thermal-mechanical and oxidation properties. J. Nucl. Mater..

[59] Salvato D., Vigier J.F., Cologna M., Luzzi L., Somers J., Tyrpekl V. (2017). Spark Plasma Sintering of fine uranium carbide powders. Ceram. Int..

[60] Alekseeva L.S., Orlova A.I., Nokhrin A.V., Boldin M.S., Lantsev E.A., Chuvil’deev V.N., Murashov A.A., Sakharov N.V. (2018). Spark Plasma Sintering of fine-grained YAG:Nd+MgO composite ceramics based on garnet-type oxide Y_2.5_Nd_0.5_Al_5_O_12_ for inert fuel matrices. Mater. Chem. Phys..

[61] Kim G., Ahn J., Ahn S. (2021). Grain growth and densification of uranium mononitride during spark plasma sintering. Ceram. Int..

[62] Yeo S., Mckenna E., Baney R., Subhash G., Tulenko J. (2013). Enhanced thermal conductivity of uranium dioxide-silicon carbide composite fuel pellets prepared by Spark Plasma Sintering (SPS). J. Nucl. Mater..

[63] Margueret A., Balice L., Popa K., Holzhäuser M., De Bona E., Bonani W., Bulgheroni A., Audubert F., Cologna M. (2022). Spark plasma sintering of UO_2_ nanopowders: Pressure, heating rate and current effects. J. Eur. Ceram. Soc..

[64] Golovkina L.S., Orlova A.I., Nokhrin A.V., Boldin M.S., Lantsev E.A., Chuvil’deev V.N., Sakharov N.V., Shotin S.V., Zelenov A.Y. (2018). Spark Plasma Sintering of fine-grained ceramic-metal composites YAG:Nd-(W,Mo) based on garnet-type oxide Y_2.5_Nd_0.5_Al_5_O_12_ for inert matrix fuel. J. Nucl. Mater..

[65] De Bona E., Balice L., Cognini L., Holzhäuser M., Popa K., Walter O., Cologna M., Prieur D., Wiss T., Baldinozzi G. (2021). Single-step, high pressure, and two-step spark plasma sintering of UO_2_ nanopowders. J. Nucl. Mater..

[66] Golovkina L.S., Orlova A.I., Chuvil’deev V.N., Boldin M.S., Lancev E.A., Nokhrin A.V., Sakharov N.V., Zelenov A.Y. (2018). Spark Plasma Sintering of high-density fine-grained Y_2.5_Nd_0.5_Al_5_O_12_+SiC composite ceramics. Mater. Res. Bull..

[67] Chen Z., Subhash G., Tulenko J.S. (2014). Master sintering curves for UO_2_ and UO_2_-SiC composite processed by spark plasma sintering. J. Nucl. Mater..

[68] Muta H., Murakami Y., Uno M., Kurosaki K., Yamanaka S. (2013). Thermophysical properties of Th_1−x_U_x_O_2_ pellets prepared by spark plasma sintering technique. J. Nucl. Sci. Techn..

[69] Golovkina L.S., Orlova A.I., Nokhrin A.V., Boldin M.S., Chuvil’deev V.N., Sakharov N.V., Belkin O.A., Shotin S.V., Zelenov A.Y. (2018). Spark Plasma Sintering of fine-grain ceramic-metal composites based on garnet-structure oxide Y_2.5_Nd_0.5_Al_5_O_12_ for Inert Matrix Fuel. Mater. Chem. Phys..

[70] O’Brien R.C., Jerred N.D. (2013). Spark Plasma Sintering of W-UO_2_ cermets. J. Nucl. Mater..

[71] Yao T., Scott S.M., Xin G., Lian J. (2016). TiO_2_ doped UO_2_ fuels sintered by spark plasma sintering. J. Nucl. Mater..

[72] Wangle T., Tyrpekl V., Cologna M., Somers J. (2015). Simulated UO_2_ fuel containing CsI by spark plasma sintering. J. Nucl. Mater..

[73] Alekseeva L.S., Nokhrin A.V., Boldin M.S., Lantsev E.A., Orlova A.I., Chuvil’deev V.N., Sakharov N.V. (2020). Fabrication of fine-grained CeO_2_-SiC ceramics for inert fuel matrices by Spark Plasma Sintering. J. Nucl. Mater..

[74] Gong B., Kardoulaki E., Yang K., Broussard A., Zhao D., Metzger K., White J.T., Sivack M.R., Mcclellan K.J., Lahoda E.J. (2022). UN and U_3_Si composites densified by spark plasma sintering for accident-tolerant fuels. Ceram. Int..

[75] Savinykh D.O., Boldin M.S., Orlova A.I., Aleksandrov A.A., Popov A.A., Murashov A.A., Nokhrin A.V., Chuvil’deev V.N., Khainakov S.A., Garcia-Granda S. (2021). Synthesis, thermal expansion behavior and sintering of sodium zirconium nickel and calcium zirconium nickel phosphates. Inorg. Mater..

[76] Savinykh D.O., Khainakov S.A., Boldin M.S., Orlova A.I., Aleksandrov A.A., Lantsev E.A., Sakharov N.V., Murashov A.A., Garcia-Granda S., Nokhrin A.V. (2018). Preparation of NZP-type Ca_0.75+0.5x_Zr_1.5_Fe_0.5_(PO_4_)_3-x_(SiO_4_)_x_ powders and ceramics, thermal expansion behavior. Inorg. Mater..

[77] Orlova A.I., Koryttseva A.K., Kanunov A.E., Chuvil’deev V.N., Moskvicheva A.V., Sakharov N.V., Boldin M.S., Nokhrin A.V. (2012). Fabrication of NaZr_2_(PO_4_)_3_-type ceramic materials by spark plasma sintering. Inorg. Mater..

[78] X-ray Interactions with Matter. https://henke.lbl.gov/optical_constants/.

[79] Yunin P.A., Nazarov A.A., Potanina E.A. (2022). Application of the GIXRD technique to investigation of damaged layers in NaNd(WO_4_)_2_ and NaNd(MoO_4_)_2_ ceramics irradiated with high-energy ions. Tech. Phys..

[80] Ziegler J.F., Ziegler M.D., Biersack J.P. (2010). SRIM—The stopping and range of ions in matter. Nucl. Instrum. Methods Phys. Res. Sect. B Beam Interact. Mater. At..

[81] Stoller R.E., Toloczko M.B., Was G.S., Certain A.G., Dwaraknath S., Garner F.A. (2013). On the use of SRIM for computing radiation damage exposure. Nucl. Instrum. Methods Phys. Res. Sect. B Beam Interact. Mater. At..

[82] Bernard-Granger G., Benameur N., Guizard C., Nygren M. (2009). Influence of graphite contamination on the optical properties of transparent spinel obtained by spark plasma sintering. Scr. Mater..

[83] Dudina D.V., Bokhonov B.B., Ukhina A.V., Anisimov A.G., Mali V.I., Esikov M.A., Batraev I.S., Kuznechik O.O., Pilinevich L.P. (2016). Reactivity of materials towards carbon of graphite foil during Spark Plasma Sintering: A case study using Ni-W powders. Mater. Lett..

[84] Wang P., Yang M., Zhang S., Tu R., Goto T., Zhang L. (2017). Suppression of carbon contamination in SPSed CaF_2_ transparent ceramics by Mo foil. J. Eur. Ceram. Soc..

[85] Kosyanov D.Y., Vornovskikh A.A., Zakharenko A.M., Gridasova E.A., Yavetskiy R.P., Dobrotvorskaya M.V., Tolmacheva A.V., Shichalin O.O., Papynov E.K., Ustinov A.Y. (2021). Influence of sintering parameters on transparency of reactive SPSed Nd^3+^:YAG ceramics. Opt. Mater..

[86] Wang P., Huang Z., Morita K., Li Q., Yang M., Zhang S., Goto T., Tu R. (2022). Influence of spark plasma sintering conditions on microstructure, carbon contamination, and transmittance of CaF_2_ ceramics. J. Eur. Ceram. Soc..

[87] Yong S.-K., Choi D.H., Lee K., Ko S.-Y., Cheong D.-I., Park Y.-J., Go S.-I. (2020). Study of the carbon contamination and carboxylate group formation in Y_2_O_3_-MgO nanocomposites fabricated by spark plasma sintering. J. Eur. Ceram. Soc..

[88] Hammoud H., Garnier V., Fantozzi G., Lachaud E., Taider S. (2019). Mechanism of carbon contamination in transparent MgAl_2_O_4_ and Y_3_Al_5_O_12_ ceramics sintered by Spark Plasma Sintering. Ceramics.

[89] Nokhrin A., Andreev P., Boldin M., Chuvil’deev V., Chegurov M., Smetanina K., Gryaznov M., Shotin S., Nazarov A., Shcherbak G. (2021). Investigation of microstructure and corrosion resistance of Ti-Al-V titanium alloys obtained by Spark Plasma Sintering. Metals.

[90] Morita K., Kim B.-N., Yoshida H., Higara K., Sakka Y. (2018). Distribution of carbon contamination in oxide ceramics occurring during spark-plasma-sintering (SPS) processing: II—Effect of SPS and loading temperatures. J. Eur. Cer. Soc..

[91] Nečina V., Pabst W. (2019). Reduction of temperature gradient and carbon contamination in electric current assisted sintering (ECAS/SPS) using a “saw-tooth” heating schedule. Ceram. Int..

[92] Bois L., Guittet M.J., Carrot F., Trocellier P., Gautier-Soyer M. (2001). Preliminary results on the leaching process of phosphate ceramics, potential host for actinide immobilization. J. Nucl. Mater..

[93] Nakata H., Kageyama T., Itoh K., Nakayama S. (2003). Leaching properties of alkali and alkaline-earth metallic elements immobilized by HZr_2_(PO_4_)_3_. J. Cer. Soc. Jpn..

[94] Nakayama S. (2022). Simultaneous immobilization of cesium and strontium by crystalline zirconium phosphate. J. Cer. Soc. Jpn..

[95] Hashimoto C., Nakayama S. (2013). Effect of treatment temperature on the immobilization of Cs and Sr to HZr_2_(PO_4_)_3_ using a autoclave. J. Nucl. Mater..

[96] Nakayama S. (2012). Immobilization of alkali, alkaline-earth and rare-earth elements by crystalline zirconium phosphate HZr_2_(PO_4_)_3_. J. Cer. Soc. Jpn..

[97] Wei Y., Luo P., Wang J., Wen J., Zhan L., Zhang X., Yang S., Wang J. (2020). Microwave-sintering preparation, phase evolution and chemical stability of Na_1-2x_Sr_x_Zr_2_(PO_4_)_3_ ceramics for immobilizing simulated radionuclides. J. Nucl. Mater..

[98] Wang J., Wei Y., Wang J., Zhang X., Wang Y., Li N. (2022). Simultaneous immobilization of radionuclides Sr and Cs by sodium zirconium phosphate type ceramics and its chemical durability. Cer. Int..

[99] Orlova A.I., Zyryanov V.N., Egor’kova O.V., Demarin V.T. (1996). Long-term hydrothermal testing of crystalline phosphates of the NZP family. Radichemistry.

[100] Orlova A.I., Zyryanov V.N., Kotel’nikov A.R. (1993). Ceramic phosphate matrices of high-level waste. Behavior under hydrothermal conditions. Radiochemistry.

[101] Kanunov A.E., Orlova A.I., Demarin V.T. (2013). Synthesis and investigation of calcium-containing phosphatosilicates with NaZr_2_(PO_4_)_3_ structure. Russ. J. Gen. Chem..

[102] Bykov D.M., Orlova A.I., Tomilin S.V., Lizin A.A., Lukinykh A.N. (2006). Americium and plutonium in trigonal phosphates (NZP type) Am_1/3_[Zr_2_(PO_4_)_3_] and Pu_1/4_[Zr_2_(PO_4_)_3_]. Radiochemistry.

[103] Nakayama S., Itoh K. (2003). Immobilization of strontium by crystalline zirconium phosphate. J. Eur. Cer. Soc..

[104] Sugantha M., Kumar N.R.S., Varadaraju U.V. (1998). Synthesis and leachability studies of NZP and eulytine phases. Waste Manag..

[105] Buvaneswari G., Varadaraju U.V. (2000). Low leachability phosphate lattices for fixation of select metal ions. Mater. Res. Bull..

[106] Pet’kov V.I., Asabina E.A., Lukuttsov A.A., Lorchemkin I.V., Alekseev A.A., Demarin V.T. (2015). Immobilization of cesium into mineral-like matrices of tridymite, kosnarite, and langbeinite structure. Radiochemistry.

[107] Shrivastava O.P., Chourasia R. (2008). Crystal chemistry of sodium zirconium phosphate based simulated ceramic waste forms of effluent cations (Ba^2+^, Sn^4+^, Fe^3+^, Cr^3+^, Ni^2+^ and Si^4+^) from light water reaction fuel reprocessing plants. J. Hazard. Mater..

[108] Kumar S.P., Gopal B. (2015). Simulated monazite crystalline wasteform La_0.4_Nd_0.1_Y_0.1_Gd_0.1_Sm_0.1_Ce_0.1_Ca_0.1_(P_0.9_Mo_0.1_O_4_): Synthesis, phase stability and chemical durability study. J. Nucl. Mater..

